# TNF-α increases the intrinsic excitability of cerebellar Purkinje cells through elevating glutamate release in Bergmann Glia

**DOI:** 10.1038/s41598-018-29786-9

**Published:** 2018-08-02

**Authors:** Hyun Geun Shim, Sung-Soo Jang, Seung Ha Kim, Eun Mi Hwang, Joo Ok Min, Hye Yun Kim, Yoo Sung Kim, Changhyeon Ryu, Geehoon Chung, YoungSoo Kim, Bo-Eun Yoon, Sang Jeong Kim

**Affiliations:** 1 Department of Physiology, Seoul National University College of Medicine, Seoul, Korea; 2 Department of Biomedical Science, Seoul National University College of Medicine, Seoul, Korea; 3 Neuroscience Research Institute, Seoul National University College of Medicine, Seoul, Korea; 4 Department of Brain and Cognitive Science, College of Science, Seoul National University, Seoul, Korea; 5 Center for Functional Connectomics, Korea Institute of Science and Technology, Seoul, Korea; 6 Department of Molecular biology, Dankook University, Chungnam, Korea; 7 Department of Pharmacy and Integrated Science and Engineering Division, Yonsei University, Incheon, Korea

## Abstract

For decades, the glial function has been highlighted not only as the ‘structural glue’, but also as an ‘active participant’ in neural circuits. Here, we suggest that tumor necrosis factor α (TNF-α), a key inflammatory cytokine, alters the neural activity of the cerebellar Purkinje cells (PCs) by facilitating gliotransmission in the juvenile male rat cerebellum. A bath application of TNF-α (100 ng/ml) in acute cerebellar slices elevates spiking activity of PCs with no alterations in the regularity of PC firings. Interestingly, the effect of TNF-α on the intrinsic excitability of PCs was abolished under a condition in which the type1 TNF receptor (TNFR1) in Bergmann glia (BG) was genetically suppressed by viral delivery of an adeno-associated virus (AAV) containing TNFR1-shRNA. In addition, we measured the concentration of glutamate derived from dissociated cerebellar cortical astrocyte cultures treated with TNF-α and observed a progressive increase of glutamate in a time-dependent manner. We hypothesised that TNF-α-induced elevation of glutamate from BGs enveloping the synaptic cleft may directly activate metabotropic glutamate receptor1 (mGluR1). Pharmacological inhibition of mGluR1, indeed, prevented the TNF-α-mediated elevation of the intrinsic excitability in PCs. Taken together, our study reveals that TNF-α triggers glutamate release in BG, thereby increasing the intrinsic excitability of cerebellar PCs in a mGluR1-dependent manner.

## Introduction

Under physiological and pathological conditions, glial cells are involved in the dynamic and/or sustained modulation of neural circuits by releasing excitatory and inhibitory gliotransmitters, such as glutamate and γ-aminobutyric acid (GABA), respectively^1–3^. The gliotransmission that primarily occurs between neurons and astrocytes contributes to Hebbian and non-Hebbian synaptic plasticity in a variety of brain regions^4–6^. In the cerebellum, PCs are surrounded by the BGs, a particular type of astrocyte in the cerebellar cortex, with processes extending into the molecular layer where parallel fibres (PFs) contact the dendrites of PCs, thereby enveloping synaptic connections^7^. Depolarising stimulation of BGs via photostimulation of BGs induces release of glutamate, thereby mediating long-term depression (LTD) at the synapse between PFs and PCs upon a mGluR1-dependent manner^2^. In addition, strong stimulation at PFs induces calcium transients within BGs, which is linked to the direct activation of mGluR1 and the α-amino-3-hydroxy-5-methyl-4-isoxazolepropionic acid receptors (AMPARs) expressed on BGs. Collectively, glutamatergic signalling from BGs into PCs is strongly implicated in cerebellar synaptic plasticity and the cerebellum-mediated behaviours^8–10^.

Immune-related cytokines such as TNF-α, interleukin-1β (IL-1β), and IL-6 have been reported to be considered as modulators in controlling the functional interactions between neurons and glial cells^11–14^. Of interest, glial TNF- α participates in synaptic adjustments against prolonged activity-deprivation in the hippocampal neurons, which is mainly mediated by the internalisation and/or insertion of AMPARs and GABA receptors (GABARs) in neurons^11,15^. In addition, exogenous application of the TNF-α triggers glutamatergic gliotransmission, resulting in the strengthening of the synaptic weight in the hippocampus^16,17^. In the cerebellum, the basal level of TNF-α is constitutively altered during the cerebellar development and abnormal changes in the concentration of TNF-α result in the manifestation of cerebellar dysfunction or diseases, indicating that TNF-α is necessary for the development of cerebellar neurons^18^. However, it is yet to be addressed what roles of TNF-α plays in modulating the intrinsic excitability of cerebellar PCs.

In the present study, we investigated the physiological effects of the TNF-α on the intrinsic excitability of PCs and whether BGs are involved in TNF-α-mediated changes of PC activity in the cerebellar circuits. Notably, TNF-α treatment in acute cerebellar slices potentiated the firing rates of the cerebellar PCs, which is thought to be mediated via the facilitation of glutamate release from the BGs. The elevation of intrinsic excitability in PCs was fully abolished by pharmacological blockage of type 1 metabotropic glutamate receptor (mGluR1), further suggesting that TNF-α-mediated an increase of excitability in PCs is required for the activation of mGluR1 in PCs.

## Results

### TNF-α treatment increases the intrinsic excitability of cerebellar PCs

**Figure 1:**
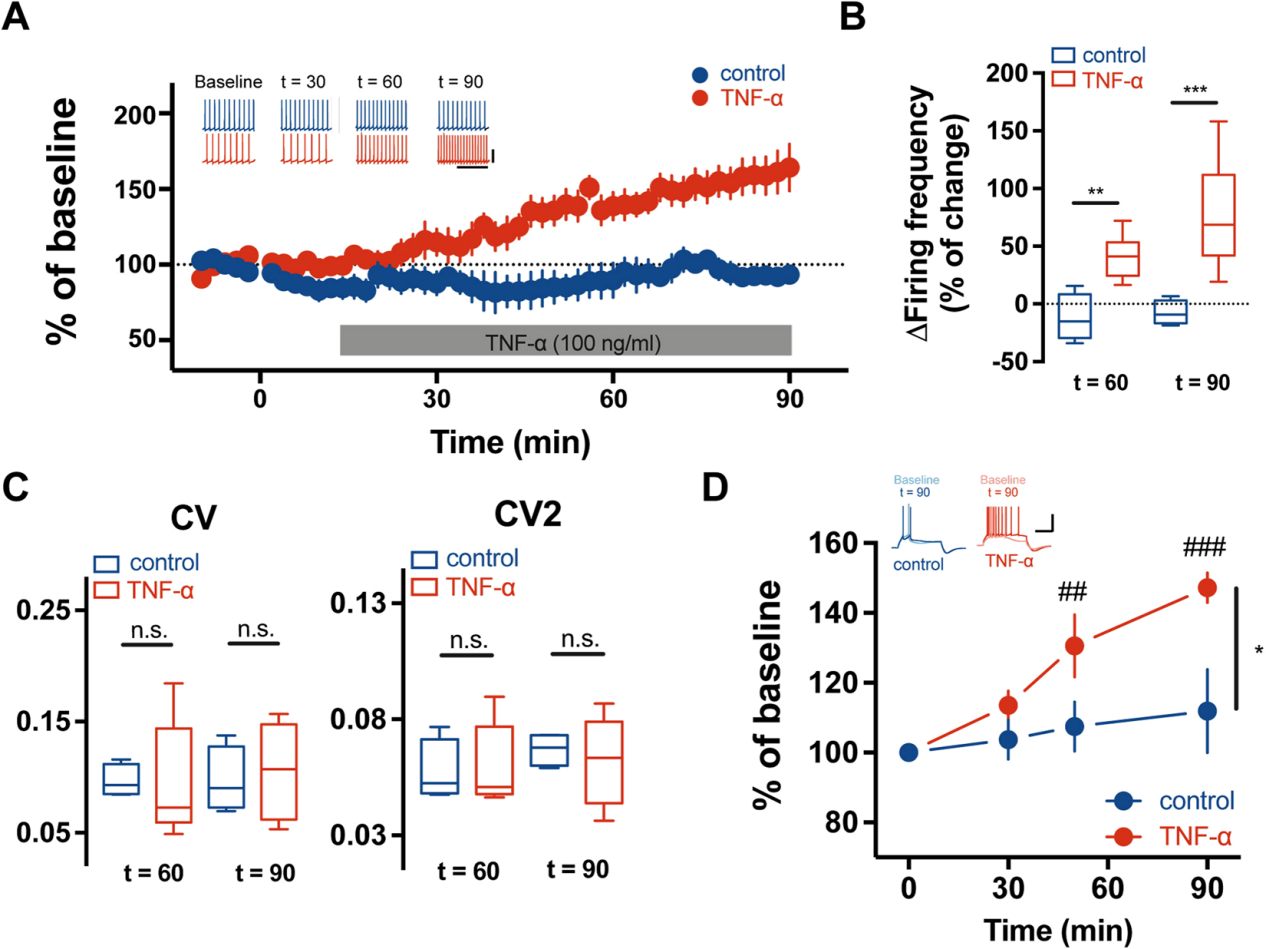
TNF-α increased the intrinsic excitability of the cerebellar PCs. (**A**) Temporal course of the spontaneous firing frequency after TNF-α application. Firing frequency are normalised for every slice by its baseline recording. TNF-α was applied at time “0” under the NBQX and picrotoxin. At least 40 min after the cytokine application, spontaneous firing frequency was increased compared to the baseline. Insets (upper) show representative traces corresponding to the control (blue) and TNF-α treated group (red) at the baseline, 30 min, 60 min and 90 min after drug application, indicating Baseline, t = 30, t = 60 and t = 90, respectively. Scale bars: 30 mV/150 ms. (**B**) Bar graphs of the percent of changes in the spontaneous firing frequency showing in (A). (**C**) Bar graphs of the coefficient variant (CV; left) and CV of adjacent intervals of the spike (CV2; right). The regularity of the spiking activity was insensitively affected by application of TNF-α. (**D**) Changes in the depolarisation-evoked spike count after TNF-α application. Spike count was normalised for every slice by its baseline recording. TNF-α was applied at time “0”. 60 min after TNF-α application, potentiation of the gain responses was elicited. Insets (left) show superimposed representative traces of evoked AP train corresponding to both groups indicated time point: t = 0 (control: light blue; TNF-α: light red) and t = 90 (control: blue; TNF-α: red). Scale bar: 20 mV/200 ms. *indicates statistical differences between groups and ^#^represents statistical difference within groups. For statistics, t-test was used for panel B and C and Two-way RM ANOVA for panel D. Post-hoc tukey test was used for different time group comparison. Error bar denotes SEM. Otherwise we note, statistical significance was indicated by *p < 0.05, **p < 0.01, ***p < 0.001; ^##^p < 0.01, ^###^p < 0.001.

To assess the effects of TNF-α on the excitability of PCs in rat cerebellar slices, we first monitored the change in the spontaneous firing activity of PCs with a bath-application of TNF-α for 90 minutes. All recordings were performed under the conditions where AMPARs and GABA_A_Rs were inhibited by 2,3-dihydroxy-6-nitro-7-sulfamoyl-benzo[f]quinoxaline-2,3-dione (NBQX, 10 μM) and picrotoxin (100 μM), respectively, in order to exclude the unexpected involvement of excitatory and inhibitory inputs in the intrinsic properties of PCs. Treatment of TNF-α significantly increased the spontaneous firing rates of the cerebellar PCs compared to baseline for 10 minutes (TNF-α; n = 6; Fig. 1Aa) whereas there were no changes in pacemaking activity from the control group (control; n = 6; Fig. 1Aa). Following the application of TNF-α, significant elevation of intrinsic excitability in PCs was exhibited at 40 minutes and maintained until 60 minutes (42.4 ± 9.1% of increase) and 90 minutes (65.2 ± 13.6% of increase), respectively (p = 0.002, t = 60; p < 0.001, t = 90; compared to control, two-sample t-test; Fig. 1Ab). However, the coefficient of variation (CV) of the spiking activity in PCs was not different between controls and TNF-α-treated cerebellar slices (con = 0.10 ± 0.01, TNF-α = 0.10 ± 0.02, t = 60; con = 0.10 ± 0.02, TNF-α = 0.11 ± 0.02, t = 90; Fig. 1B). The CV of adjacent intervals of the spike (CV2) was 0.07 ± 0.003 in control groups versus 0.06 ± 0.010 in the TNF-α treated groups at t = 90 (Fig. 1B), suggesting that the regularity of the spiking activity in PCs was not affected by the TNF-α treatment. To further investigate whether the TNF-α treatment affects evoked action potential (AP) firings of PCs, we measured the number of spikes evoked by the somatic depolarising current injection. In parallel with elevated spontaneous firing rates, evoked AP firings of PCs were significantly increased 60 minutes after incubation with TNF-α [n = 4; 113.6 ± 5.6% of baseline in t = 30, p = 0.32, two-way Repeated Measured (RM) ANOVA Tukey post-hoc test compared to the value in t = 0; 130.5 ± 4.1% in t = 60, p = 0.005; 147.2 ± 4.3% in t = 90, p < 0.001; Fig. 1C] whereas the number of spikes was not changed during the entire recording time in the absence of TNF-α (n = 5).

**Figure 2:**
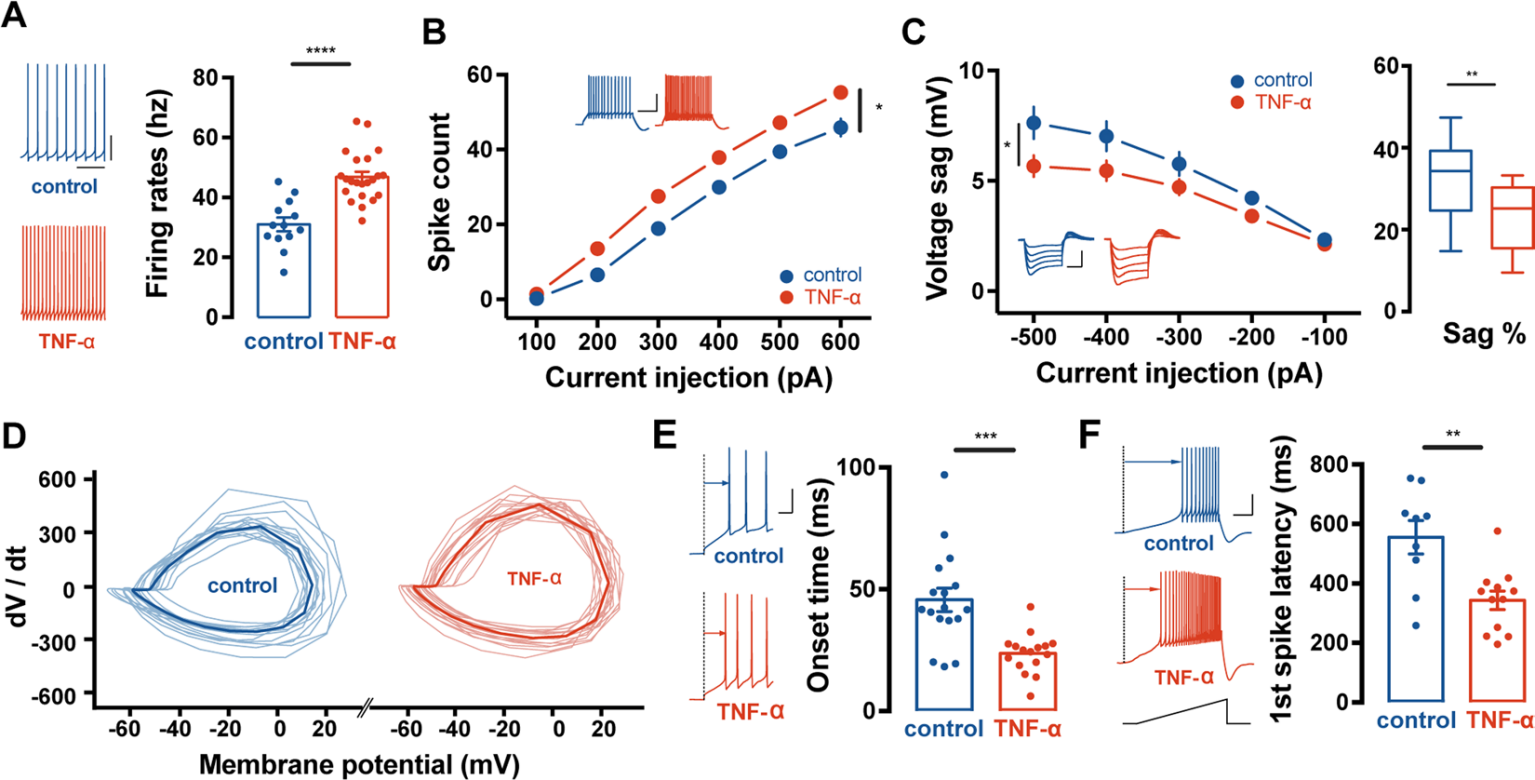
Pre-incubation with TNF-α altered the intrinsic properties of the cerebellar PCs. (**A**) Bar graphs of the spontaneous firing frequency after pre-incubation with TNF-α for 60 min. Compared to control (blue), TNF-α treated neurons (red) show higher firing frequency. Insets (left) show representative traces of spontaneous firing activity from both groups. Scale bar: 20 mV/40 ms. (**B**) Plots of the input current-spike count relationship from control and TNF-α treated neurons. Pre-incubation with TNF-α increases gain responses of the cerebellar PCs in response to square-wised current injection ranging from 100 pA to 600 pA for 500 ms. Insets (left upper) show representative traces of evoked AP trains. Scale bar: 20 mv/200 ms. (**C**) Plots of the sag potential in response to hyperpolarising current injection ranging from −100 pA to −500 pA for 500 ms (middle) and recalculated percent of sag potential from control and TNF-α treated neurons. TNF-α reduced sag potential and percent of sag potential of the PCs. Insets (left) show representative traces of voltage deflections. Scale bar: 20 mV/200 ms. (**D**) Phase plot of the single AP from control (left) and TNF-α treated PCs (right). TNF-α treated neurons show more rapid changes in the dV/dt corresponding membrane potential but peak amplitude and the voltage threshold for AP generation are not significantly different between groups. (**E**) Superimposed traces of single AP from control (blue) and TNF-α treated PCs (red). Scale bar: 20 mV/0.5 ms. (**F**) Bar graphs of spike onset time of the first AP elicited by depolarising current injection. Pre-incubation with TNF-α reduces delay to the onset time. Inset shows representative traces of control (blue) and TNF-α treated PC (red). Scale bar: 20 mV/10 ms. (**G**) Bar graphs of firing frequency and the first spike latency eliciting by ramp current injection (400 pA s−1 for 1 s). TNF-α (red) reduces the time delay for spike generation compared to the control (blue). Insets show representative traces of the ramp current-elicited AP train from both groups. Scale bas: 20 mV/200 ms. For statistics, t-test was used for panel A, C (right), F and G and Two-way RM ANOVA for panel B and C (middle). Error bar denotes SEM.

Next, we pre-incubated slices with TNF-α for 60 minutes to rule out the possibility that a long-lasting whole-cell patch clamp configuration could cause a washout of intracellular fluid. Similar to our observation as described in Fig. 1, spontaneous firing rates were significantly increased in PCs under the condition in which slices were pre-incubated with TNF-α (control: 31.0 ± 2.3, n = 13; TNF-α: 46.0 ± 1.6, n = 21; p < 0.001, t-test; Fig. 2A). Although the voltage threshold did not significantly differ between the two groups (Supplemental Table 1), depolarisation-evoked AP firings (control: n = 17; TNF-α: n = 16; F = 7.115, p = 0.014, two-way RM ANOVA; Fig. 2B) and input resistances (Supplemental Table 1) of PCs were increased in PCs with pre-incubation of TNF-α, implying that TNF-α gives rise to larger voltage deflection in response to membrane depolarization and thereby results in augmentation of evoked AP firings of PCs. In addition, we measured voltage sag by injecting square-wised hyperpolarising step of current injection from −100 to −500 pA for 500 ms. Based on the differences of maximal voltage deflection in response to hyperpolarising current input between groups, membrane potential was normalised by the maximal negative voltage and then recalculated as a percent. Pre-incubation with TNF-α resulted in a reduction in the voltage sag (control: n = 13; TNF-α: n = 19; F = 4.580, p = 0.041, two-way RM ANOVA; Fig. 2C left) and sag % (control = 32.5 ± 2.9%, n = 12; TNF-α = 23.1 ± 1.7, n = 19, p = 0.006, t-test; Fig. 2C right) of PCs. Furthermore, we confirmed that TNF-α-induced an elevation of intrinsic excitability in PCs from the organotypic cultured slices where the level of immune responses is minimised to prevent unexpected effects from immune molecules produced during the process of brain slice preparation process. In parallel with the data shown in Fig. 2, the spike count was increased among the most of the ranges of the current steps (control: n = 30; TNF-α: n = 33, F = 17.4, p < 0.001) and the input resistance was markedly enhanced as well (control = 93.0 ± 2.5 MΩ, n = 31; TNF-α = 108.1 ± 1 MΩ, n = 39, p = 0.001, t-test; Supplemental Fig. 1A–C) after pre-incubation with TNF- α for 60 minutes. The voltage sag was also decreased in the TNF-α-incubated PCs compared to the controls (control n = 21; TNF- α n = 12, F = 10.9, p = 0.0024, two-way RM ANOVA; Supplemental Fig. 1D). Phase analysis of the APs recorded from TNF-α-incubated slices displayed faster kinetics to reach the maximal amplitude compared to those from the control (Fig. 2D, Supplemental Table 1). The AP rise time was robustly decreased (control = 0.19 ± 0.008; TNF-α = 0.16 ± 0.004, p = 0.001, t-test) and the AP half-width (full width of half maximum, FWHM) was reduced after TNF-α application (control = 0.32 ± 0.01; TNF-α = 0.28 ± 0.01, p = 0.014, t-test; Supplemental Table 1). In addition, we determined if TNF-α affects onset time delay of the first AP by injecting depolarising currents and ramp current (400 pA s^−1^ for 1 s; Fig. 2F,G; referred as the AP onset time and the first-spike latency, respectively). We found that both onset time and the first spike latency displayed a significant reduction in the TNF-α incubated PCs compared with the PCs in the control (Fig. 2E,F). Detailed parameters of the AP properties are summarised in the Supplemental Table 1. Furthermore, we asked if TNF-α would permanently and irreversibly lead to the alteration of excitability in PCs. To answer this, we washed out TNF- α from the pre-incubated-cerebellar slices for 2-3 hrs by putting slices in the normal artificial cerebrospinal fluid (aCSF) and then assessed the excitability of PCs. Compared with the value from shown in TNF-α-treated neurons previously shown (Fig. 2), spontaneous firing rates and evoked spike counts were not altered after the TNF- α washed-out (Supplemental Fig. 2A,B). In addition, onset time and first-spike latency were similar to the value obtained from the TNF- α-incubated slices as shown (Supplemental Fig. 1C,D). Therefore, we concluded that exogenous TNF- α triggers plasticity of the intrinsic excitability from cerebellar PCs, which appears to be irreversible.

### BGs are involved in TNF-α-induced potentiation of the intrinsic excitability of PCs via elevation of glutamate release

**Figure 3:**
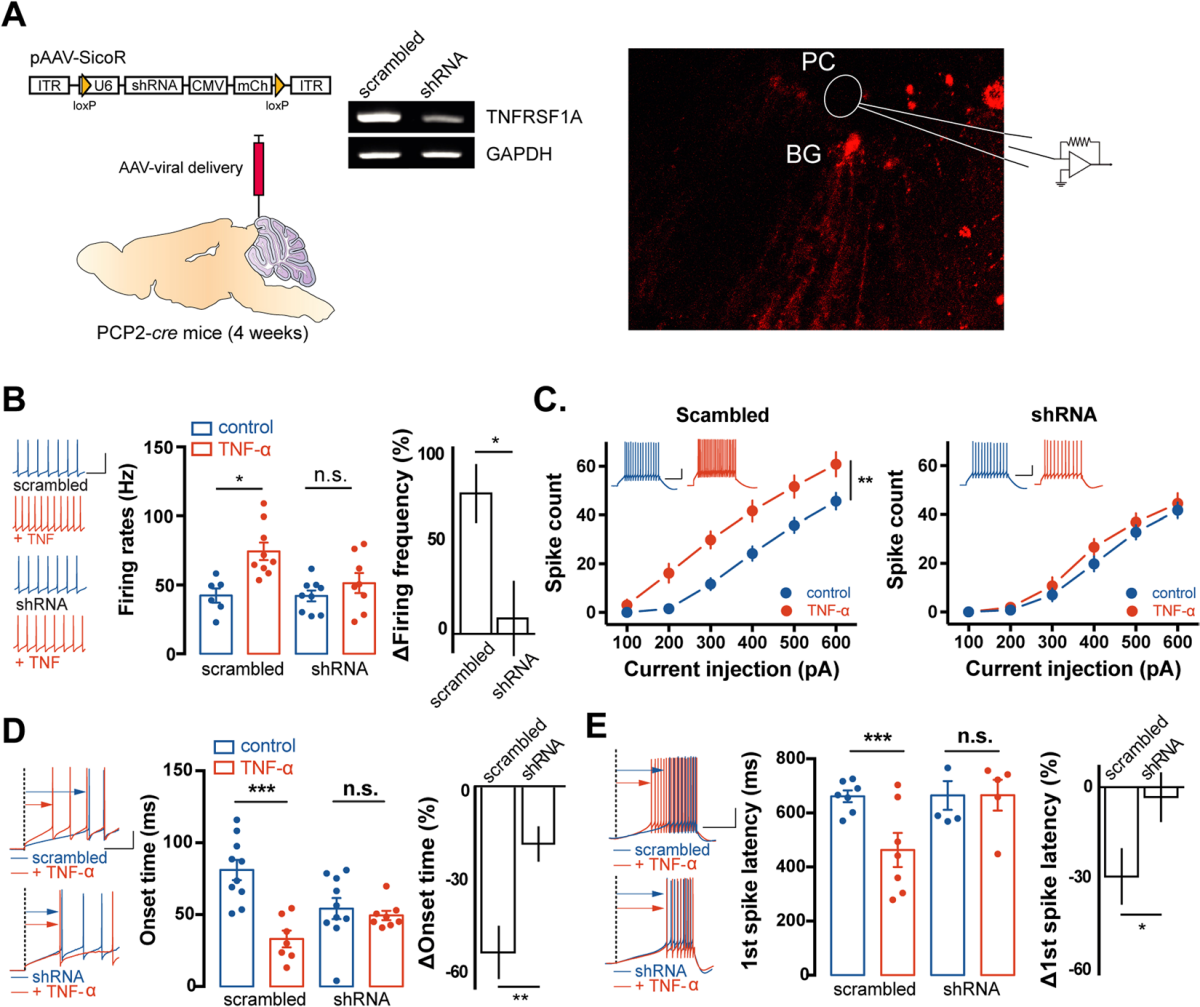
Interference of TNFR1 in BGs suppresses the effect of the TNF-α on the PC excitability. (**A**) Scheme of the model (left) and example images of conditional TNFR1 knock-down (right). Viral vector contains shRNA for *tnfr1* gene interference inserted within loxP site and delivered to mice expressing cre recombinase under the PCP2 promoter (PCP2-*cre* mice). Example gels show downregulation of tnfr1 gene expressing (right upper). The representative gels were cropped from two different gels (full-length gel is presented in Supplementary figure 2). Confocal image (right below) indicates that cell-type specific expression of TNFR1-shRNA in Bergmann glia, tagging by mCherry fluorescence (right below). Electrophysiological recordings are performed in PCs around mCherry-tagged BGs. (**B**) Bar graphs of the spontaneous firing frequency after pre-incubation with TNF-α for 60 min (middle) and its percent changes in firing frequency (right). Value from mean firing frequency after TNF-α treatment was normalised by control value (right). Scrambled virus is used as control (blue) of virus containing shRNA (red), potentiation of spontaneous firing frequency was suppressed by interference TNFR1. Insets show representative traces of spontaneous spiking activity from each groups. Scale bar: 40 mV/40 ms. (**C**) Plots of the excitability after pre-incubation with TNF-α in shRNA containing virus-delivered slices (left). Depolarisation-evoked spike count increased after pre-incubation with TNF-α in (left) in scrambled virus-delivered slices, but the effects of TNF-α on the excitability were prevented by inhibition of TNFR1 expression (right). Insets show representative traces of evoked AP train from control (blue) and TNF-α (red) treated neuron in each groups. Scale bar: 20 mV/200 ms. (**D**) Bar graphs of spike onset time after pre-incubation of TNF-α from control and shRNA-containing virus-delivered slices (middle) and its percent changes (right). Value from mean time of delay to onset AP after TNF-α treatment was normalised by control value (right). Interference of TNFR1 prevented the reduction of onset time by TNF-α. Insets show superimposed representative traces of delay to onset of spike generation corresponding the control (blue) and TNF-α (red, upper: scrambled; below: shRNA). Scale bar: 20 mV/10 ms. (**E**) Bar graphs of the first spike latency after pre-incubation of TNF-α from control and shRNA-containing virus-delivered slices (middle) and its percent changes (right). Value from mean time to elicit the first spike responding to ramp current injection after TNF-α treatment was normalised by control value (right). Interference of TNFR1 prevented the reduction of the first spike latency by TNF-α. Insets show superimposed representative traces of the ramp current-elicited AP train corresponding the control (blue) and TNF-α (red, upper: scrambled; below: shRNA). 20 mV/200 ms. For statistics, t-test was used for panel B, D and E and Two-way RM ANOVA for panel C (plots). Error bar denotes SEM.

It has widely been reported that TNF-α modulates neural activity via the activation of glial cells^11,16^. To investigate whether TNF-α induced potentiation of PC intrinsic excitability is associated with glial cells in the cerebellum, we inhibited the expression of the type 1 TNF receptor (TNFR1) in BGs in a cell-type specific manner using a molecular genetic strategy. A viral vector approach with an AAV using the pSicoR system (Addgene, Cambridge, MA, USA) containing a TNFR1-shRNA was generated^19^ and then delivered into a *cre* recombinase expressing mice line in PCs (PCP2-*cre*) (Fig. 3A). In this strategy, an shRNA for the interference of the *tnfr1* gene is inserted within the loxP site, so that the shRNA is not expressed in the cre-expressing cells. Thus, transfected cells displayed mCherry-fluorescence, indicating the suppression of TNFR1. When we delivered TNFR1-shRNA to the cerebellar cortex in the lobular 3–5 region of PCP2-*cre* mice, mCherry fluorescence was observed in BGs, identified by their morphology and localisation, within the PC layer. For the whole-cell patch clamp recording with PCs in acute cerebellar slices, PCs located around mCherry-expressing cells were selected. In TNFR1-shRNA transduced slices, TNF-α-induced potentiation of spontaneous spiking activity of PCs was fully abolished [Δfiring rates: scrambled (%) = 75.8 ± 15.0, n = 9; TNFR1-shRNA = 9.6 ± 19.4, n = 4, p = 0.03, t-test; Fig. 3B]. In addition, neither the spike count (scrambled: control n = 10, TNF-α n = 7, F = 8.688, p = 0.026; TNFR1-shRNA: control n = 10, TNF-α n = 11, F = 0.42, p = 0.533, two-way RM ANOVA; Fig. 3C left) nor the input resistance was changed after TNF-a treatment in the TNFR1-shRNA transduced group (Supplemental Table 2). Furthermore, TNFR1 expression interference with shRNA resulted in the suppression of the TNF-α-mediated effects on the onset time [Δonset time (%): scrambled = −52.6 ± 8.3, n = 7; shRNA = −17.3 ± 5.4, n = 8, p = 0.003, t-test; Fig. 3D] and on the first-spike latency [Δfirst spike latency (%): scrambled = −30 ± 9.5, n = 7; shRNA = −2.2 ± 8.4, n = 5, p = 0.03, t-test; Fig. 3E]. Taken together, we suggest that the potentiation of PC intrinsic excitability after TNF-α treatment requires activation of glial TNFR1.

### Glutamate transporters are not affected by TNF-α application

**Figure 4:**
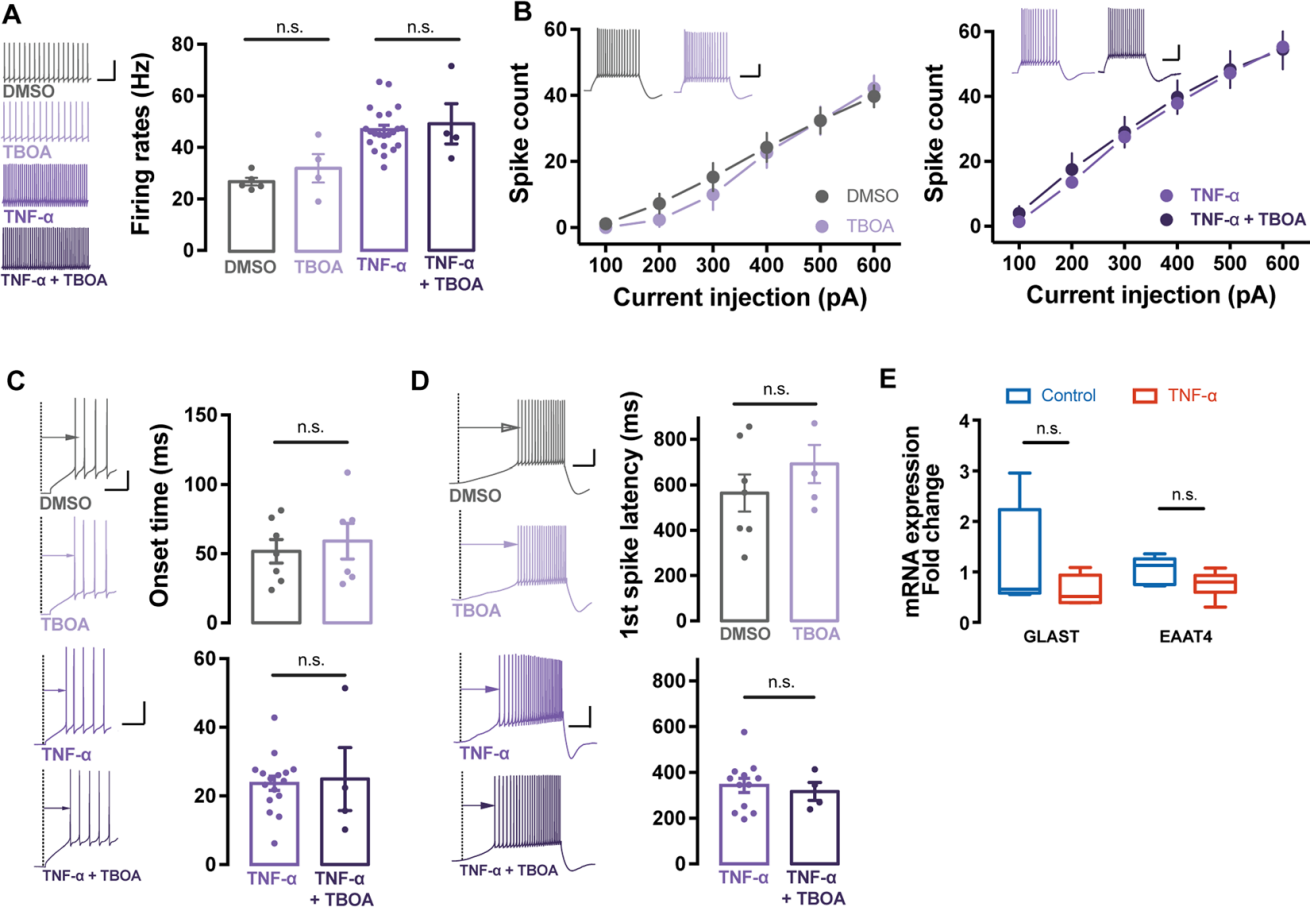
Suppression of the glutamate transporters are not involved in the TNF-α-induced intrinsic plasticity of the cerebellar PCs. (**A**) Bar graph of spontaneous firing rates (Hz) after application of TBOA (light purple) comparing to the DMSO control (grey) and TNF-α (purple) comparing to TNF-α and TBOA treated neurons (dark purple). There no significant differences between groups (DMSO vs. TBOA; TNF-α vs. TNF-α + TBOA), indicating that inhibition of glutamate uptake is not involved in the changes of intrinsic excitability. Inset shows representative traces of spontaneous spiking activity from both group (left). Scale bar: 10 mV/200 ms. (**B**) Plots of the input current-spike count relationship from application of DMSO (grey) vs. TBOA (light purple) (left) and TNF-α treated neurons (purple) vs. co-treatment of TBOA + TNF-α (dark purple) (right). Gain responses of the cerebellar PCs is not be affected by TBOA. Insets (left upper) show representative traces of evoked AP trains. Scale bar: 20 mV/200 ms. (**C**) Bar graphs of spike onset time of the first AP elicited by depolarising current injection. Delay to the onset time is not affected by application of TBOA. Inset shows representative traces of DMSO (grey), TBOA (light purple), TNF-α (purple) and co-treatment of TNF-α and TBOA (dark purple). Scale bar: 20 mV/50 ms. (**D**) Bar graphs of the first spike latency eliciting by ramp current injection (400 pA s−1 for 1 s). Time delay for spike generation is not changed by TBOA treatment. Insets show representative traces of the ramp current-elicited AP train from DMSO (grey), TBOA (light purple), TNF-α (purple) and co-treatment of TNF-α and TBOA group (dark purple). Scale bar: 20 mV/50 ms. (**E**) Box and whiskers chart of changes of mRNA expression level of glutamate transporters (GLAST and EAAT4). Application of TNF-α tend to attenuate mRNA level of glutamate transporters,however, there were no significant alterations of mRNA level compared to control and TNF-α-treated neurons. For statistics, Mann-Whitney test was used for panal E and t-test was used for panel A, C and D and Two-way RM ANOVA for panel B. n.s. means statistical non-significant. Error bar denotes SEM.

Based on a previous report that TNF-α inhibits the activity of the glutamate transporter expressed in glia^20^, we tested if TNF-α effects on the intrinsic excitability of PCs are derived from the suppression of glutamate transporters in the cerebellar cortex. To answer this, we applied L-threo-beta-enzyloxyaspartate (TBOA), a potent blocker of the excitatory amino acid transporter, to the cerebellar slices and then monitored the excitability of PCs under the presence of excitatory and inhibitory synaptic transmission inhibitors. Notably, the excitability of PCs was not changed by pharmacological inhibition of glutamate transporters (Fig. 4A–D), indicating that the inhibition of glutamate transporters does not result in the alteration of PC excitability. Next, we aimed to further investigate whether the suppression of these transporters could strengthens TNF-α-induced elevation of the excitability in PCs. Our data revealed no further effect of TNF-α by application of TBOA, implying that the activity of glutamate transporters may not be involved in the TNF-α-induced intrinsic plasticity of PCs. (Fig. 4A–D). In addition, we additionally tested if the expressional levels of glutamate transporters was suppressed by the application of TNF-α. To prove this, we compared the relative expressional level of mRNA encoding to the glutamate aspartate transporter (GLAST, EAAT1) and the excitatory amino acid transporter 4 (EAAT4), which are dominantly expressed in BG and PCs, respectively. Between the control and TNF-α-treated group, using quantitative real-time polymerase chain reaction (qRT-PCR), we found that TNF-α did not affect the mRNA levels of glutamate transporters (GLAST: p = 0.21; EAAT4: p = 0.22, Mann-Whitney test, Fig. 4E). Therefore, we neglect the possibility of which TNF-α-induced intrinsic plasticity was derived from inhibition of glutamate transporters expressed in BG.

### TNF-α increases intrinsic excitability of the cerebellar PCs in an mGluR1-dependent manner

**Figure 5:**
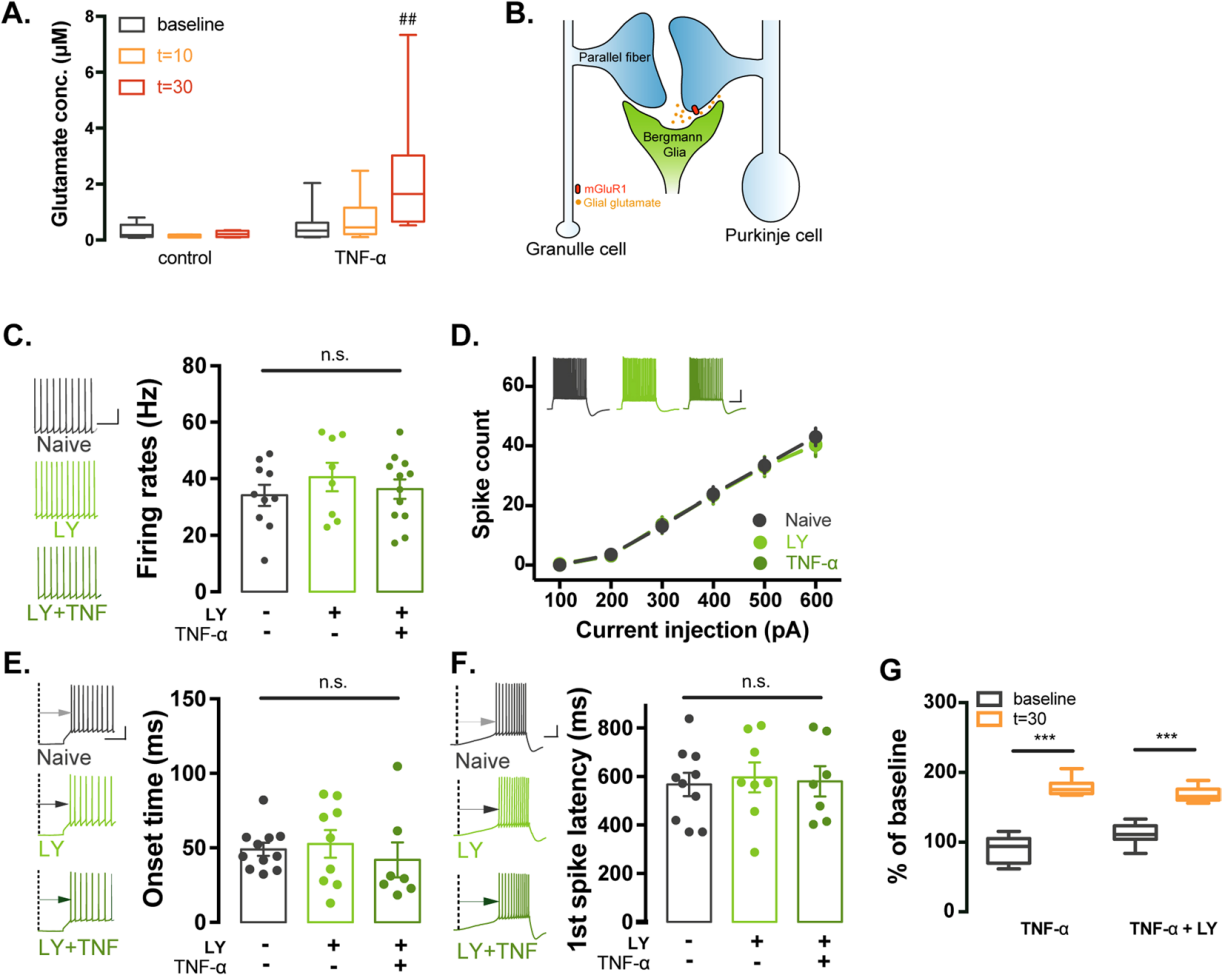
TNF-α-induced potentiation of intrinsic excitability of PCs requires mGluR1 activation. (**A**) Bar graphs of HPLC analysis for glutamate concentration. BGs release glutamate corresponding to treatment of TNF-α into the cerebellar astrocyte cultures in a time-dependent manner. Glutamate concentration was increased 30 min after the application of TNF-α compared to the value from “t = 0” (right). In the control experiment (left), the concentration of glutamate is not changed. *indicates statistical differences between groups (t-test) and ^#^represents statistical difference comparing to the value at t = 0 (One-way ANOVA on Ranks test, post-hoc tukey test). (**B**) Illustration of proposed model for the mechanism by which TNF-α increases the intrinsic excitability of PCs. Exogenous TNF-α binds to TNFR1 expressed in BG and the glutamatergic gliotransmission consequently is facilitated. These processes result in the accumulation of glutamate in the synaptic cleft thereby activation of mGluR1 in PC and the intrinsic excitability is potentiated. (**C**) Bar graphs of the spontaneous firing frequency after pre-incubation with TNF-α for 60 min in presence of selective mGluR1 inhibitor, LY 367385 (LY). Pre-incubation with LY (light green) or TNF-α + LY (TNF + LY, green) showed no alteration of spontaneous firing frequency compared to the control groups (control: no drugs, dark grey). Insets (left) show representative traces of spontaneous firing corresponding to the control, LY and LY + TNF group. Scale bar: 20 mV/40 ms. (**D**) Plots of the input current-spike count relationship from control (no drug), LY and TNF + LY group. Application of mGluR1 inhibitor (LY) blocked the effect of TNF-α on the excitability of the cerebellar PCs. Insets (upper) show representative traces of evoked AP trains. Scale bar: 20 mv/200 ms. (**E**) Bar graphs of spike onset time of the first AP elicited by depolarising current injection. LY prevented reduction of delay to the onset time by TNF-α. Inset (left) shows representative traces. Scale bar: 20 mV/10 ms. (**F**) Bar graphs of firing frequency and the first spike latency eliciting by ramp current injection (400 pA s−1 for 1 s). LY prevented reduction of the time delay for spike generation by TNF-α. Insets show representative traces of the ramp current-elicited AP train from. Scale bas: 20 mV/200 ms. (**G**) Bar graphs of changes of glutamate concentration from HPLC analysis. BGs release glutamate corresponding to treatment of TNF-α into the cerebellar astrocyte cultures and its effect is not affected by inhibition of mGluR1. Glutamate concentration is increased 30 min after the application of TNF-α compared to the value from baseline (left, yellow) and there are no significant changes of glutamate concentration after LY367385 treatment with TNF-α (right). Asterisks represent statistical difference comparing to the value at baseline. For statistics, One-way ANOVA was used for panel A, C and E and Mann-Whitney test was used for panel G. ^#^denotes statistical significance by post-hoc tukey test using for comparison within groups for panel A. ^##^And ^***^indicate p < 0.005 and p < 0.001, respectively and n.s. means statistical non-significant. Error bar denotes SEM.

Previous reports have described that neurons communicate with surrounding glial cells through gliotransmitter such as glutamate and ATP^8,21^. Of particular note, TNF-α triggers the release of neurotransmitters from glia, which influences on the neural activity^17^. In addition, it has been widely demonstrated that BGs envelop PC synapses and release neurotransmitter within the ectopic site of the PC synapses, contributing to a spillover of neurotransmitters near these synaptic loci^2^. Therefore, we asked if TNF-α could modulate glutamatergic gliotransmission from BGs. To address this, the concentration of glutamate was measured by high-performance liquid chromatography (HPLC) in the cerebellar astrocyte cultures following application of TNF-α. Our data revealed that glutamate concentration was increased in a time-dependent manner and a significant change was detected in 30 minutes after TNF-α treatment [0.52 ± 0.10 μM (t = 0, n = 8) vs. 2.28 ± 0.81 μM (t = 30, n = 8), q = 3.925, p < 0.05, one-way ANOVA on Ranks post-hoc Tukey test; Fig. 5A]. Considering their adjacency between BGs and PCs, this finding led us to hypothesise that released glutamate from BGs is likely to promote activation of mGluR1 expressed in the perisynaptic area of PC dendrites which may underlie TNF-α-induced intrinsic plasticity of PCs (Fig. 5B). To confirm our speculation, we treated a selective mGluR1 inhibitor, LY 367385 with TNF-α and measured evoked AP firings of PCs. Interestingly, pharmacological inhibition of mGluR1 prevented the effects of TNF-α on the intrinsic excitability of PCs (Fig. 5C,D), suggesting that TNF-α induced elevation of intrinsic excitability of PCs was dependent on the mGluR1 activity. In parallel with these changes, there were no significant differences in the onset time, the first spike latency, fAHP, and sAHP in APs and input resistance between two groups in a presence of the mGluR1 inhibitor (Fig. 5E,F and Supplemental Table 3). To exclude the possibility that the application of LY 367385 affected the mGluR1α expression in BGs, the glutamate concentration was measured by HPLC analysis following application of TNF-α and co-treatment of TNF-α with LY 367385 onto the cerebellar astrocyte culture. We observed that application of LY 367385 with TNF-α showed no further changes in glutamate concentration compared to the value obtained from the TNF-α-treated group [TNF-α: n = 10 (t = 30); LY 367385 + TNF-α: n = 10 (t = 30), p = 0.566, Two-way ANOVA post-hoc tukey test; Fig. 5G]. Taken together, out results suggest that facilitation of glutamatergic gliotransmission via exogenous TNF-α treatment potentiates the intrinsic excitability of cerebellar PCs in an mGluR1-dependent manner.

## Discussion

Neuro-glia interactions are known to be crucial for the functional modulation of cerebellar behaviours^10^. However, the underlying mechanism are less understood. Here, we provided insights into how TNF-α regulates the excitability of PCs in cerebellar circuits. In this study, we found that TNF-α facilitates glutamatergic gliotransmission, leading in turn to the potentiation of intrinsic excitability in cerebellar PCs. When expression of TNFR1 in BGs was inhibited in a cell-type specific manner, the TNF-α-induced intrinsic plasticity of PCs was abolished, implying an involvement of BG activity in potentiation of PC excitability after TNF-α treatment. Strikingly, in a cerebellar astrocyte culture, incubation of TNF-α gave rise to an increase of glutamate, thus we suggest that TNF-α-induced intrinsic plasticity of PCs is facilitated via glutamate release from BGs. Furthermore, the alterations in active properties and frequency of PC firing following TNF-α application was prevented by pharmacological inhibition of mGluR1 and the mGluR1-mediated current was augmented after TNF-α treatment. Taken together, our results propose a plausible mechanism by which BGs contribute to the modulation of the intrinsic excitability of PCs in the cerebellum.

Previous reports have shown that BGs contribute to the modulation of PC activity via gliotransmission^22–24^. Photostimulation of BGs can trigger glutamate release onto PCs, giving a rise to mGluR1-dependent LTD between PF-PC synapses and induction of the horizontal optokinetic reflex upon repetitive visual stimuli^2^. Our observation that genetic suppression of TNFR1 in BGs does not induce TNF-α-mediated elevation of intrinsic excitability in PCs (Fig. 3) suggests that communication between PCs and BGs requires TNF-α as a mediator. Given the anatomical feature of the tripartite synapses in the cerebellar cortex, glutamate released from BGs is likely to activate mGluR1 expressed in the perisynaptic area of the cerebellar PCs. Here, we presented experimental evidence showing that the mGluR1 expressed in PCs are required for neuro-glia interaction mediated by on the exogenous TNF-α. Potentiation of the intrinsic excitability of PCs by TNF-α showed a non-reversible permanent alteration within the time window we analysed here. In parallel with this result, it has previously been described that the activation of group1 mGluRs induces irreversible changes of neural activity in the hippocampus^25,26^. Furthermore, the alteration of the spiking activity in PCs after TNF-α application was abolished by mGluR1 antagonist (Fig. 4). Collectively, exogenous TNF-α may enhance the neuro-glia interactions leading to the maintenance of the sufficient glutamate levels in the synaptic cleft to activate mGluR1 and thereby induce neuronal plasticity.

TNF-α-mediated glutamatergic gliotransmission may require a variety of cellular events. Previous reports described that TNF-α activates NF-κB signalling involved in suppression of glutamate transporter expression in glia leading to inhibition of glutamate uptake^27^, eventually increasing the amount of glutamate at the synapses. However, our data did not show any alterations in mRNA levels of GLAST and EAAT4 following application of TNF-α (Fig. 4E). In addition, pharmacological inhibition of glutamate transporter using TBOA did not change the excitability of PCs (Fig. 4A–D), indicating there is an alternative mechanism by which TNF-α potentiates gliotransmission in the cerebellar cortex that needs to be addressed. Previous studies have suggested a possible mechanism of glutamate release from astrocytes by exogenous TNF-α. The activation of microglia elevates the level of the soluble TNF-α that binds TNFR1 in astrocytes, which in turn leads to glutamate release via the increase of prostaglandin synthesis^16,28^, implying that the TNF-α enhances efficacy of glutamate release from glia to glia and neurons. In addition, TNF-α also promotes vesicle docking in the astrocytes resulting in the augmentation of glutamate release^11^. Alternatively, TNF-α also increases in the glutamate synthesis in the glia. A previous study described that TNF-α triggers the up-regulation of the glutamate converting enzyme, glutaminase, from glial cells^27^. Considering that our data shows an increase in glutamate release from cerebellar astrocytes at 30 minutes after TNF-α application, transcription and/or translation may be involved in facilitating glutamate release from BG. Collectively, it would be plausible that TNF-α strengthens gliotransmission by elevating the rates of glutamate production and/or release from glial cells, which in turn increases the amount of ambient glutamate at tripartite synapses where PCs, BGs, and PFs are integrated. Further studies should address the downstream mechanisms by which TNF-α elevates the amount of glutamate derived from BGs.

Activation of mGluR1 enables te persistent change in neuronal excitability as well as the induction of synaptic plasticity through the conductance alterations in a variety of ion channels^29^. In the current study, the robust alterations of input resistance and voltage sag in TNF-α-treated neurons (Supplemental Table 1 and Fig. 2C) strongly suggest that hyperpolarisation-activated cyclic nucleotide-gated (HCN) channels are a plausible candidate for these changes^26,30,31^, despite the fact that HCN channels are not involved in the regulation of spontaneous firing rates in the cerebellar PCs^32,33^. Large conductance Ca^2+^-activated potassium (BK) channels could also be a contributors to the mGluR1-dependent intrinsic plasticity by TNF-α based on the literatures reporting that mGluR controls the BK gating and modulate the pacemaking activity^34,35^. Small conductance Ca^2+^-activated potassium (SK) channels could be one mechanism for the TNF-α-induced intrinsic plasticity because activity-dependent intrinsic plasticity is modulated by SK channel activity^36^. In addition, considering our observations from this study, including the reduction of spike onset time, and latency, D-type potassium channels might also be required for the TNF-α-induced intrinsic plasticity in PCs^37–39^. Collectively, TNF-α-induced intrinsic plasticity might involve synergistic alterations of several ion channels that modulate neural excitability^40^, nevertheless detailed mechanisms by which activation of the BG affects the PCs activity need to be further investigated.

Previous implications have shown that the alterations of the levels of TNF-α in the serum and/or in the cerebrospinal fluid are associated with various brain diseases in human^41,42^. For example, pro-inflammatory cytokines including TNF-α have been reported to be elevated in psychiatric disorder patients^43^. The concentration of TNF-α we used in this study (100 ng/ml) was quite high, which is likely to reflect a more pathophysiological circumstance. This concentration could lead to an imbalance of AMPA/GABA receptor activity at dendrites of neurons and give rise to inappropriate expression of Ca^2+^-permeable AMPA receptors not only at synapses but also in extrasynaptic sites^44,45^ which in turn could result in excitotoxicity. In addition, local inflammation can triggers abnormal strengthening of synaptic weight through TNF-α, consequently causing an impairment in memory function^46^. Therefore, the *TNFR1* gene has been considered to be an attractive therapeutic target for reducing AMPA receptor-mediated excitotoxicity^47^. In contrast, TNF-α serves as protective and adaptive cytokine for regulating the homeostasis of the neural network activity^48,49^. Of interest, ablation of TNF signalling shows more severe neurological impairment and demyelination with high mortality in animal models such as autoimmune diseases and brain injury^50^. Although it remains unclear whether the amount of TNF-α (100 ng/ml) used in our study represents physiological conditions, a certain amount of TNF-α is indeed necessary for stabilising neural excitability in the brain. Thus, further investigation on how TNF-α affects cerebellar network activity has to be addressed. Taken together, TNF-α may act as a functional switch that not only determines the magnitude of basal neural activity, but also regulates the functional homeostasis of the cerebellum.

What is the functional consequence of TNF-α-induced intrinsic plasticity of PCs in the cerebellar cortex? Elevation of TNF-α concentration in the cerebellum has been implicated in cerebellar pathophysiological dysfunction^51,52^. The data presented here shows electrophysiological outcomes of TNF-α elevation in cerebellar circuits. Alteration of PC spiking activity indeed has been implicated in the underlying mechanisms of neurodegenerative diseases and seizure generation^53,54^. Thus, the TNF-α-induced potentiation of excitability might reflect several cerebellum-related diseases. In a different pathological viewpoint, TNF-α-induced elevated excitability in PCs may undergo homeostatic adjustments of the cerebellar network by decreasing a potential increase in the deep cerebellar nuclei against neuronal hyper-excitability upon brain inflammation. We previously reported that chronic alteration of cerebellar activity leads to homeostatic changes in the intrinsic excitability of PCs, suggesting that an increase of TNF-α could serve adaptive functions in the cerebellum^30^. TNF-α also plays a neurotrophic role in the differentiation of PCs at the early developmental stages^18^. In addition, given the note that prolonged optic-stimulation of PCs facilitates elimination of climbing fibres (CFs)^55^, elevation of excitability in PCs through TNF-α could be involved in the fine-tuning of cerebellar development. Lastly, the fact that glutamate released from BGs is involved in PF-PC LTD and regulation of motor behaviour suggests that glutamatergic gliotransmission may be critical for neural plasticity in the cerebellum^2,10^. Here, we demonstrated that TNF-α facilitates glutamate spillover which enables the activation of mGluR1 leading to induce the neural plasticity in PCs. Further studies should address how elevation of TNF-α in the cerebellum contributes to synaptic and intrinsic plasticity in the cerebellar cortex and influences cerebellum-associated behaviours.

## Material and Methods

### Animals and Slice Preparations

All animal use was in accordance with protocols approved by the Institution’s Animal Care and Use Committee of Seoul National University College of Medicine. Cerebellar parasagittal slices were dissected into 250 μm by vibratome (VT1200, Leica, Germany) from anaesthetized juvenile rats (P17–25) and 6 weeks old male C57BL/6 mice in ice-cold standard artificial cerebrospinal fluid (aCSF) containing the following (in mM): 125 NaCl, 2.5 KCl, 1 MgCl_2_, 2 CaCl_2_, 1.25 NaH_2_PO_4_, 26 NaHCO_3_, 10 glucose bubbled with 95% O_2_ and 5% CO_2_. For recovery, slices were incubated at 32 °C for 30 minutes and further 1 hour further at room temperature.

### Electrophysiology

Slices were put onto a submerged recording chamber on the stage of an Olympus microscope (BX50WI, Japan) and perfused with aCSF, and kept in place with a nylon-strung platinum anchor. All recordings were performed using the multiclamp 700B patch-clamp amplifier (Axon Instruments) with a sampling frequency of 20 kHz and signals were filtered at 2 kHz. To exclude the influence of the synaptic activity on the intrinsic properties of neurons, excitatory and inhibitory synaptic inputs were totally blocked with 10 μM NBQX and 100 μM picrotoxin, respectively. Patch pipettes (3-4 MΩ) were borosilicate glass and filled with internal solution containing the following: 9 KCl, 10 KOH, 120 K-gluconate, 3.48 MgCl_2_, 10 HEPES, 4 NaCl, 4 Na_2_ATP, 0.4 Na_3_GTP and 17.5 sucrose (pH 7.25) for current clamp recordings. The membrane potential was held at −70 mV in voltage-clamp (VC) and current-clamp mode (CC). When the series resistance (R_s_) varied by >15% and the injection current for the holding potential exceeded 500 pA, recordings were discarded. To evaluate the PC excitability, we monitored the spontaneous spiking activity with no current injection from the membrane potential of each neurons and/or measured the spike counts elicited by the injection of square-wised a series of current steps ranging from +100 pA to +600 pA with increments of 100 pA, and with a step interval of 4.5 s for 500 ms from a membrane potential of −70 mV in CC mode.

### Production of TNFR1 shRNA-pSicoR Adeno-Associated Virus

The TNFRSF1A nucleotides from 385 to 405(5′-GGAATTATTCTTTGGATGGGC-3′; NM_013091) were selected for the target region of TNFR1 shRNA. For the suppression of TNFR1 expression in BG cells in a cell-type specific manner, we first cloned TNFR1 shRNA into pSicoR-mChe-empty lentiviral vector(addgene #21907) as previously reported^22^. Next, U6-loxP-CMV-mCherry-loxP sequence was amplified by PCR with XbaI(forward) or BglII(reverse)-containing specific primers, and then it was sub-cloned into the pAAV-MCS vector(Stratagene). Recombinant AAV was produced by co-transfection into HEK293A cells using AAV-DJ Helper-Free Packaging System (Cellbiolabs).

### Cerebellar Astrocyte Cultures

Cerebellar cortices were dissected from P0–P1 postnatal mice, cleared of adherent meninges, minced, and dissociated into a single-cell suspension by trituration. Dissociated cells were plated onto 12-mm glass coverslips coated with 0.1 mg/ml poly D-lysine. Cells were grown in DMEM supplemented with 25 mM glucose, 10% heat-inactivated horse serum, 10% heat-inactivated fetal bovine serum, 2 mM glutamine, and 1000 units/ml penicillin-streptomycin. Cultures were maintained at 37 °C in humidified atmosphere of 5% CO_2_.

### HPLC

Prior to HPLC assay anlaysis, astrocytes were washed 3 times with PBS. Cells were incubated with TNF-α (100 ng/ml) or PBS in time course (0 min, 10 min, 30 min and 90 min). The amino acid content was derivatized with o-phthaldialdehyde (OPA) and quantified with UV (DAD) detection. OPA-derivatized samples were collected with a programmed autosampler and injected onto a Zorbax Eclipse Plus C18 column with detection at 338 nm (reference, 390 nm). Mobile phase A was 40 mM Na2HPO4 (pH 7.8) and phase B was acetonitrile–methanol–water (45:45:10, v/v/v). The flow rate was 2 ml/min with a gradient condition that allowed for 1.9 min at 0% B and a rise to 26% B over a 12.5-min step. Subsequent washing at 100% B and equilibration at 0% B was performed within a total retention time of 15 min. Reagents for OPA derivatization and all equipment for HPLC analysis were obtained from Agilent Technologies.

### Real-time Quantitative PCR (qPCR)

Sampling. Sagittal slices of the cerebellar vermis (300 µm thick) were obtained from juvenile rats (postnatal day 20–22). For extracting total RNA, we used RNeasy Mini Kit (QIAGEN) and followed manufacturer instructions. DNase I (QIAGEN) was treated. A total of 350 ng of total RNA per sample was reverse transcribed into cDNA using SuperScript III First-Strand (Invitrogen). qPCR was performed using the CFX Connect system (Bio-Rad) with SYBR Premix (TaKaRa). qPCR reactions with three individual pairs of primers were specific for: rat GLAST, forward primer, TGTCTTCTCCATGTGCTTCG, reverse primer, CAAGAAGAGGATGCCCAGAG, rat EAAT4, forward primer, GGAGACTGTGCCTGTACCTGG, reverse primer, GCAGAGCTGGAAGAGGTACCC, rat GAPDH, forward primer, CCACGAAGGGTGGAGCCAAA, reverse primer, GTCTTCTGGGTGGCAGTGATGG.

All primers were previously used^56,57^. The mRNA expression levels of TNF-α-treated neurons relative to control were normalized according to the ∆Ct method. All ∆Ct values were normalized by Ct value of GAPDH.

### Data Acquisition and Analysis

All data were acquired by the Clampex software (Molecular Devices) and analysed by IgorPro 8.1 (Wavemetrics). Electrical properties of the neurons were monitored with following parameters: R_S_ was calculated by fitting a single exponentials to the voltage responses of the test pulse (−5 mV); Input resistance (R_in_) was determined from negative peak voltage deflection in response to brief hyperpolarising current injection (−100 pA; 100 ms); Voltage threshold (V_threshold_) of AP was defined as the voltage where the dV/dt first exceeds 30–60 mV/ms); Fast afterhyperpolarization (fAHP) and medium afterhyperpolarization (mAHP) were calculated the difference between voltage threshold and hyperpolarised negative peak voltage after the first AP or depolarising square current injection, respectively. The AP waveform, including AP amplitude, half-width (FWHM) and 10–90% rise time was analysed from the first evoked AP of the firing train when +300 pA of the depolarising current was injected; AP amplitude was determi ned as difference between peak amplitude and the voltage threshold of the AP. Half-width and 10–90% rise time were the time duration at the half-maximal voltage, elevation time from 10 to 90% of the maximal AP voltage, respectively. The spike onset time was determined as the delay from the beginning point of the depolarising current injection to the voltage threshold where the upstroke phase of the first spike was initiated when the 300 pA of current was injected. The first spike latency was evaluated by measuring the delay from beginning point of ramp current injection (400 pA s^−1^ for 1 s).

Data were presented as the mean ± SEM and statistical evaluations were performed using the normality test, equal variant test, independent *t*-test, One-way repetitive measured (RM) ANOVA and Two-way RM ANOVA with post *hoc* tukey test by Origin 8.5, SigmaPlot 12.0 and Prism 7.0 software. The sample size was approved by power analysis using the G*power 3.1.9.2.

## Electronic supplementary material

Supplemental information
