# Supplementary material for: TNF-α increases the intrinsic excitability of cerebellar Purkinje cells through elevating glutamate release in Bergmann Glia
Source: Sci Rep. 2018 Aug 2;8:11589. doi: 10.1038/s41598-018-29786-9 (PMC6072779; doi:10.1038/s41598-018-29786-9)
Supplement: Supplementary file 1 — Supplemental information [file 41598_2018_29786_MOESM1_ESM.docx]

**Title:**

**TNF-α increases the intrinsic excitability of cerebellar Purkinje cells through elevating glutamate release in Bergmann Glia**

**TNF-α increases the intrinsic excitability of cerebellar Purkinje cells through elevating glutamate release in Bergmann Glia**

Hyun Geun Shim^1,2†^, Sung-Soo Jang^1,3†^, Seung Ha Kim^1,2^, Eun Mi Hwang^5^, Joo Ok Min^6^, Hye Yun Kim^7^, Yoo Sung Kim^6^, Changhyeon Ryu^1,2,3^, Geehoon Chung^1,4^, YoungSoo Kim^7^, Bo-Eun Yoon^6^ and Sang Jeong Kim^1,2,3*^

^1^Department of Physiology, Seoul National University College of Medicine, Seoul, Korea.

^2^Department of Biomedical Science, Seoul National University College of Medicine, Seoul, Korea.

^3^Neuroscience Research Institute, Seoul National University College of Medicine, Seoul, Korea.

^4^Department of Brain and Cognitive Science, College of Science, Seoul National University, Seoul, Korea

^5^Center for Functional Connectomics, Korea Institute of Science and Technology, Seoul, Korea

^6^Department of Molecular biology, Dankook University, Chungnam, Korea.

^7^Department of Pharmacy and Integrated Science and Engineering Division, Yonsei University, Incheon, Korea.

^*^ These authors contributed equally to this work.

^†^To whom correspondence should be addressed: Department of Physiology, Seoul National University College of Medicine, 103 Daehak-ro, Jongro-gu, Seoul, 03087, South Korea. Tel.: +82-2-740-8229; E-mail: [sangjkim@snu.ac.kr](mailto:sangjkim@snu.ac.kr)

**Corresponding Author**

Sang Jeong Kim

Department of Physiology, Seoul National University College of Medicine, 03087 Seoul, Republic of Korea

[sangjkim@snu.ac.kr](mailto:sangjkim@snu.ac.kr)

Supplementary information

Index

1. Supplementary figure 1. (related to fig 2)
2. Supplementary table 1. (related to fig 2)
3. Supplementary figure 2 (related to fig 2)
4. Supplementary table 2. (related to fig 3)
5. Supplementary figure 3 (related to fig 3)
6. Supplementary table 3. (related to fig 5)
7. Method


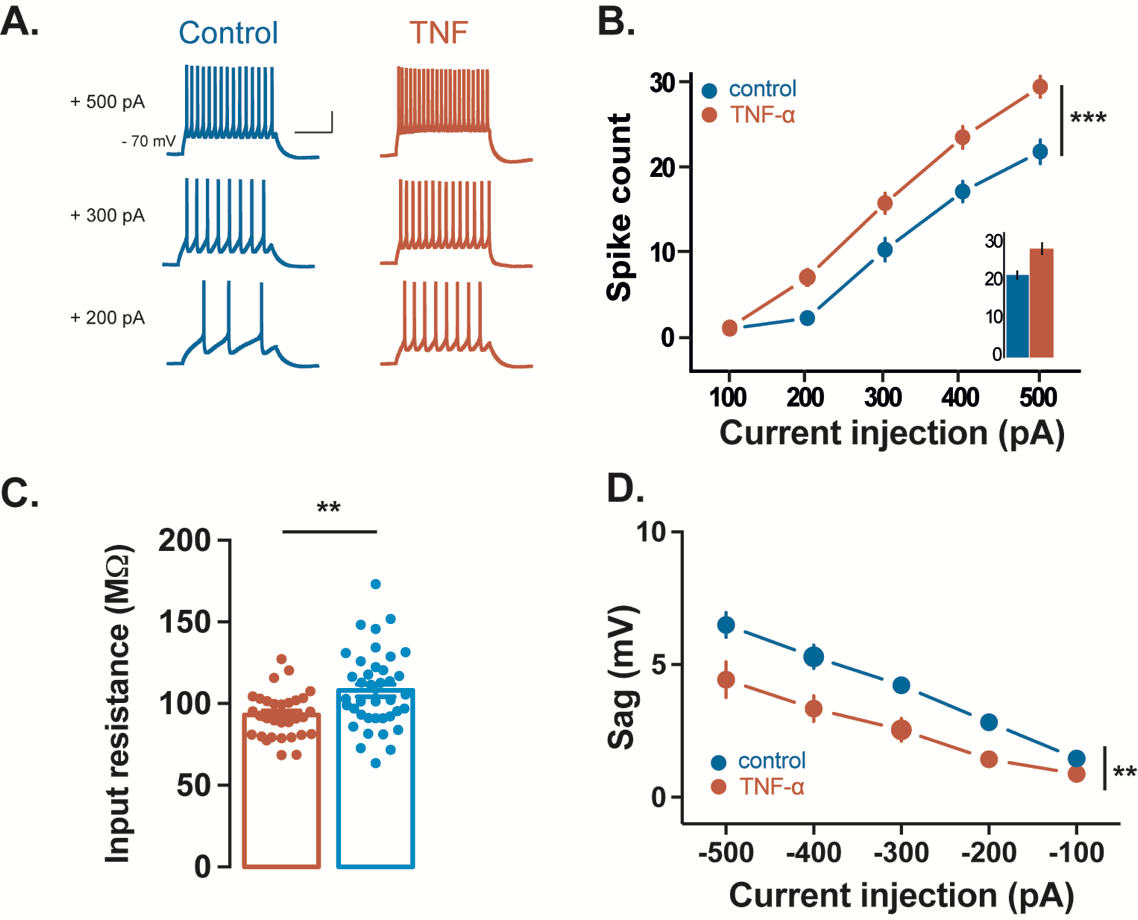


Supplementary figure 1 (related to fig 2). TNF-α increased intrinsic excitability of the cerebellar Purkinje cells from organotypic culture.

1. Representative traces of evoked spike train from organotypic cerebellar slices corresponding to the square-wised somatic current injection in control (left) and TNF-α incubation (right) group.
2. Plots of excitability after pre-incubation with TNF-α in control (blue) and TNF-α treated neurons (red). Inset (right bottom) shows bar graph of the number of spikes at 500 pA of current injection.
3. Bar graphs of input resistance from TNF-α-treated neurons (red) comparing to the control (blue).
4. Plots of sag potential after pre-incubation (red) comparing to the control (blue). Insets show representative traces of voltage deflection in response to the square-wised hyperpolarising step current injection.

For statistics, Two-way RM ANOVA test was used for panal B and D and t-test was used for C. Asterisks denotes the statistical significance: **p<0.01, ***p<0.0001. Error bar indicates SEM.

|  |  | **Control**  **(n = 17)** | | |  |  | **TNF-α (n = 16)** | | |
| --- | --- | --- | --- | --- | --- | --- | --- | --- | --- |
|  |  |  |  |  |  |  |  |  |  |
| V_threshold_ (mV) |  | -44.8 | ± | 1.0 |  |  | -43.5 | ± | 1.3 |
| AP amplitude (mV) |  | 66.9 | ± | 1.2 |  |  | 69.1 | ± | 1.2 |
| fAHP (mV) |  | 10.8 | ± | 0.8 |  |  | 10.7 | ± | 1.1 |
| mAHP (mV) |  | 10.0 | ± | 0.9 |  |  | 8.6 | ± | 0.9 |
| Rise time ** (ms) |  | 0.19 | ± | 0.008 |  |  | 0.16 | ± | 0.004 |
| Decay time (ms) |  | 0.23 | ± | 0.01 |  |  | 0.20 | ± | 0.010 |
| FWHM * (ms) |  | 0.32 | ± | 0.01 |  |  | 0.28 | ± | 0.01 |
| Onset time *** (ms) |  | 45.7 | ± | 4.8 |  |  | 22.9 | ± | 2.1 |
| 1st spike latency ** (ms) |  | 554.8 | ± | 56.3 |  |  | 343.2 | ± | 31.0 |
| Input resistance * (MΩ) |  | 72.2 | ± | 3.6 |  |  | 89.5 | ± | 6.3 |

Supplementary Table 1 (related to fig 2). Active properties of the cerebellar PCs after incubation with the TNF-α. Asterisks denotes the statistical significance: * p < 0.05; ** p < 0.01; *** p < 0.001, t-test. Values are presented by Mean ± S.E.M.


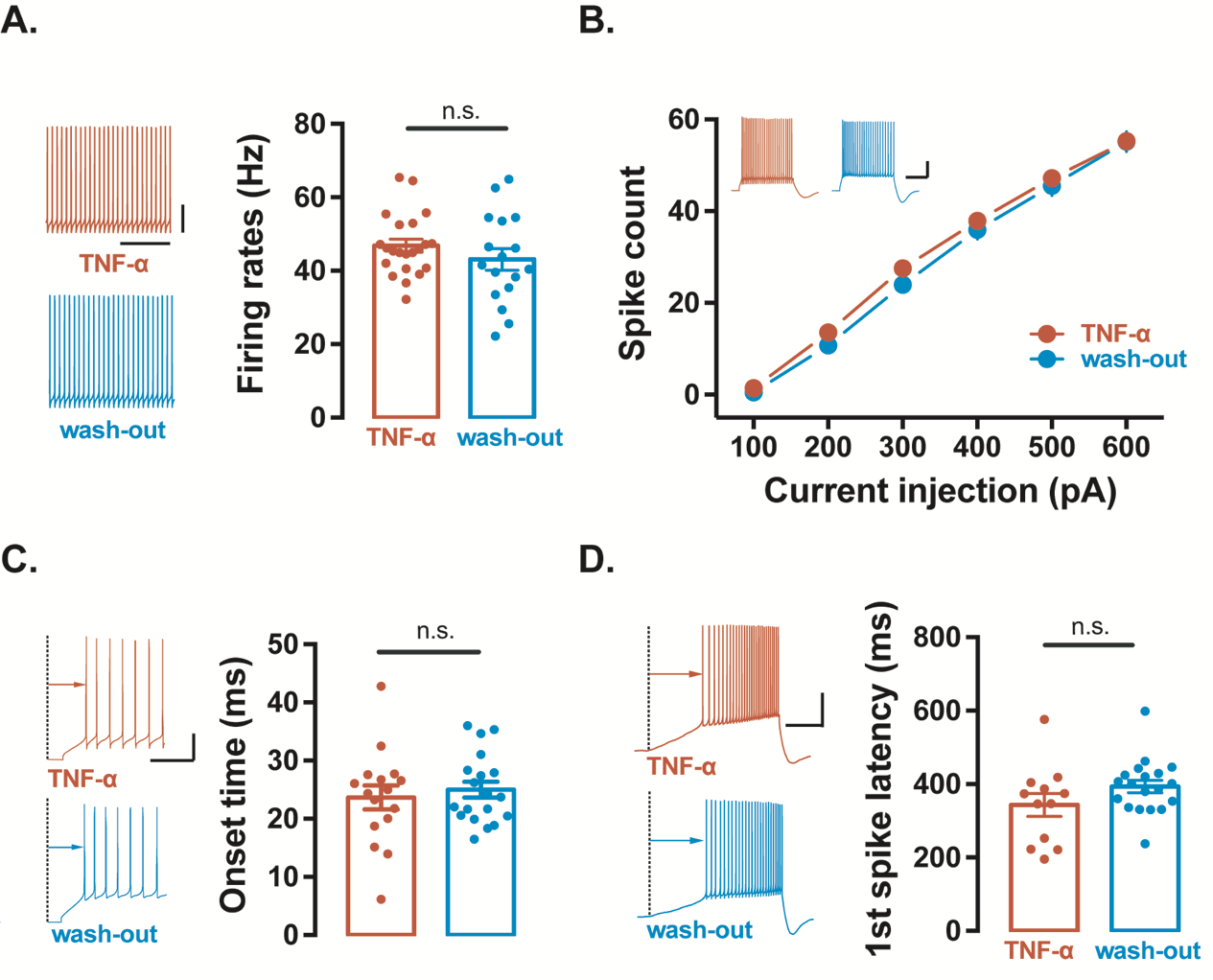


Supplementary figure 2 (related to fig. 2). TNF-α induced permanent changes in the excitability of cerebellar PCs.

1. Bar graph of spontaneous firing rates (Hz) after wash-out TNF-α for 2 – 3 hrs (blue) comparing to the value in which pre-incubation with TNF-α shown in fig 2A (red). There no significant differences between groups, indicating that TNF-α triggers plastic changes of intrinsic excitability. Inset shows representative traces of spontaneous spiking activity from both group (left).
2. Plots of the input current-spike count relationship from TNF-α treated neurons and after wash-out from TNF-α. TNF-α-induced potentiation of gain responses of the cerebellar PCs is not be attenuated by washout for 2 – 3 hrs. Insets (left upper) show representative traces of evoked AP trains. Scale bar: 20 mv / 200 ms
3. Bar graphs of spike onset time of the first AP elicited by depolarising current injection. Effect of pre-incubation with TNF-α on the delay to the onset time is not affected by washout from TNF-α. Inset shows representative traces of TNF-α-treated neurons (red) and the value after washout (blue). Scale bar: 20 mV / 10 ms.
4. Bar graphs of firing frequency and the first spike latency eliciting by ramp current injection (400 pA s−1 for 1s). Time delay for spike generation shown in TNF-α-treated neurons (red) is not changed after washout for 2 – 3 hrs (blue). Insets show representative traces of the ramp current-elicited AP train from both groups. Scale bas: 20 mV / 200 ms.

For statistics, t-test was used for A, C and D. n.s. denotes non-significant. Error bar indicates SEM.

|  |  | **Scrambled** | | | | | | | | | | | | **TNFR1-shRNA** | | | | | | | | | | | | |
| --- | --- | --- | --- | --- | --- | --- | --- | --- | --- | --- | --- | --- | --- | --- | --- | --- | --- | --- | --- | --- | --- | --- | --- | --- | --- | --- |
|  |  | Control (n=9) | | | |  | |  | | TNF-α (n=7) | | | | Control (n=10) | | | |  | |  | | TNF-α (n=9) | | | |  |
| V_threshold_ (mV) |  | -44.6 | ± | 0.9 |  | |  | | -44.2 | | ± | 1.7 | -42.3 | | ± | 1.6 |  | |  | | -44.4 | | ± | 1.1 |  |  |
| AP amplitude (mV) |  | 65.4 | ± | 1.6 |  | |  | | 62.7 | | ± | 2.0 | 62.5 | | ± | 2.8 |  | |  | | 63.2 | | ± | 1.9 |  |  |
| fAHP (mV) |  | 13.2 | ± | 0.9 |  | |  | | 14.1 | | ± | 0.9 | 15.4 | | ± | 0.5 |  | |  | | 14.1 | | ± | 1.3 |  |  |
| mAHP (mV) |  | 6.4 | ± | 0.5 |  | |  | | 3.0 | | ± | 0.6 *** | 9.7 | | ± | 0.5 |  | |  | | 5.9 | | ± | 0.9 ** |  |  |
| Rise time (ms) |  | 0.14 | ± | 0.003 |  | |  | | 0.18 | | ± | 0.032 | 0.16 | | ± | 0.003 |  | |  | | 0.17 | | ± | 0.017 |  |  |
| Decay time (ms) |  | 0.17 | ± | 0.012 |  | |  | | 0.19 | | ± | 0.012 | 0.17 | | ± | 0.006 |  | |  | | 0.21 | | ± | 0.032 |  |  |
| FWHM (ms) |  | 0.24 | ± | 0.011 |  | |  | | 0.27 | | ± | 0.019 | 0.24 | | ± | 0.007 |  | |  | | 0.29 | | ± | 0.039 |  |  |
| Onset time (ms) |  | 81.04 | ± | 7.013 |  | |  | | 33.11 | | ± | 5.8 *** | 54.20 | | ± | 7.300 |  | |  | | 45.40 | | ± | 4.958 |  |  |
| 1st spike latency (ms) |  | 661.3 | ± | 21.8 |  | |  | | 462.8 | | ± | 63.1 * | 680.7 | | ± | 39.2 |  | |  | | 665.5 | | ± | 56.9 |  |  |
| Input resistance (MΩ) |  | 50.4 | ± | 9.2 |  | |  | | 74.5 | | ± | 6.4 * | 42.1 | | ± | 3.9 |  | |  | | 56.8 | | ± | 8.5 |  |  |

Supplementary Table 2 (related to fig 3). Active properties of the cerebellar PCs after cell-type specific suppression of TNFR1. Asterisks denotes the statistical significance: * p < 0.05; ** p < 0.01; *** p < 0.001, t-test. Values are presented by Mean ± S.E.M.


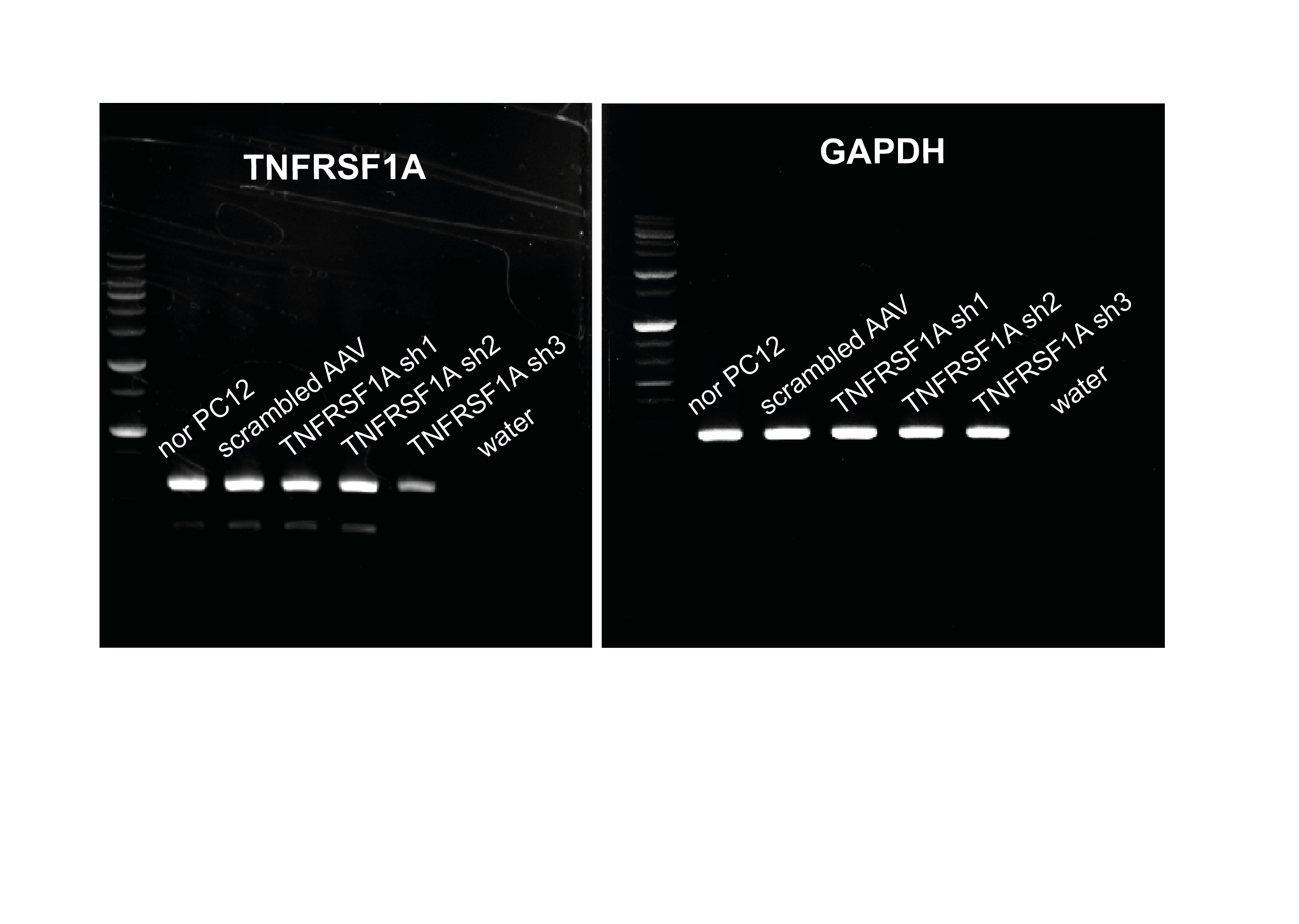


Supplementary figure 3 (related to fig. 3). Full-length gels for assessment of suppression of *tnfr1* gene expression after delivery of TNFR1-shRNA (left) and GAPDH control (right). mRNA level of TNFR1 was measured from normal PC12 cells (nor PC12) and different variant of *TNFR1*-shRNA were verified. In this study, we used TNFRSF1A sh3, and its targeting sequence is described in the *Material and Method.*

|  |  | mGluR1 inhibition | | | | | | | | | | |
| --- | --- | --- | --- | --- | --- | --- | --- | --- | --- | --- | --- | --- |
|  |  | Control (n=11) | | |  | LY 367380 (n=9) | | |  | LY 367380 + TNF-α (n=7) | | |
| V_threshold_ (mV) |  | -45.8 | ± | 0.6 |  | -43.9 | ± | 1.7 |  | -44.4 | ± | 2.2 |
| AP amplitude (mV) |  | 71.5 | ± | 1.8 |  | 67.1 | ± | 1.8 |  | 65.8 | ± | 3.3 |
| fAHP (mV) |  | 8.2 | ± | 0.7 |  | 11.7 | ± | 1.3 |  | 9.1 | ± | 1.4 |
| mAHP (mV) |  | 10.1 | ± | 0.8 |  | 9.0 | ± | 1.2 |  | 11.4 | ± | 1.0 |
| Rise time (ms) |  | 0.17 | ± | 0.009 |  | 0.17 | ± | 0.008 |  | 0.19 | ± | 0.015 |
| Decay time ^#^  (ms) |  | 0.22 | ± | 0.005 |  | 0.20 | ± | 0.009 * |  | 0.26 | ± | 0.021 |
| FWHM ^#^ (ms) |  | 0.30 | ± | 0.009 |  | 0.29 | ± | 0.010 * |  | 0.36 | ± | 0.034 |
| Onset time (ms) |  | 49.02 | ± | 4.327 |  | 52.66 | ± | 9.248 |  | 41.93 | ± | 11.737 |
| 1st spike latency (ms) |  | 567.2 | ± | 47.9 |  | 596.0 | ± | 61.8 |  | 580.6 | ± | 62.4 |
| Input resistance (MΩ) |  | 68.7 | ± | 5.7 |  | 62.2 | ± | 2.6 |  | 61.9 | ± | 3.7 |

Supplementary Table 3 (related to fig 5). Active properties of the cerebellar PCs after pharmacological inhibition of mGluR1 (LY 367380). # denotes statistical significance by One-way ANOVA analysis and asterisk denote statistical significance by post-hoc tukey test using for comparison within groups: ^#^ p < 0.05, * p < 0.05.

Material and method

*Organotypic slice culture*

All procedure was approved by the Institution’s Animal Care and Use Committee of Seoul National University College of Medicine. Cerebellar sagittal slices were dissected into 250 μm by vibratome from anaesthetized P10 rats in ice-cold standard artificial cerebrospinal fluid (aCSF) contained with the following (in mM): 124 NaCl, 2.5 KCl, 1 NaH2PO4, 1.3 MgCl2, 2.5 CaCl2, 26.2 NaHCO3 and 20 D-glucose, saturated with 95 % O2, and 5 % CO2. Sagittal planes of cerebellar slices were transferred onto membrane of culture insert (pore size 0.4 µm) in 6-well-plastic plates. Culture medium (1 ml), composed of 50 % basal medium with Earle’s salts, 25 % HBSS, 25 % Heat-inactivated horse serum, 1 % L-glutaMaxTM-1 and 5 mg/ml glucose, was added into each well below the culture inserts. Cultured slices were incubated at 35°C in an atmosphere of humidified 5 % CO2, and half of medium was replaced every 2 – 3 days.
